# Energy metabolism dysregulation in idiopathic inflammatory myopathies: mechanisms and therapeutic implications

**DOI:** 10.3389/fimmu.2026.1781434

**Published:** 2026-04-17

**Authors:** Yuan Fang, Huihui Chi, Jialin Teng, Chengde Yang, Yue Sun, Yutong Su

**Affiliations:** 1 Department of Rheumatology and Immunology, Ruijin Hospital, Shanghai Jiao Tong University School of Medicine, Shanghai, China; 2 Shanghai Hospital of Civil Aviation Administration of China, Shanghai, China

**Keywords:** energy metabolism, idiopathic inflammatory myopathies, immunometabolism, metabolic reprogramming, mitochondrial dysfunction, oxidative stress

## Abstract

Idiopathic inflammatory myopathies (IIMs) are being increasingly recognized as disorders driven by profound disturbances in cellular energy metabolism rather than inflammation alone. Recent studies have highlighted mitochondrial dysfunction, oxidative stress, and metabolic reprogramming across glucose, lipid, and amino acid pathways as central mechanisms linking energy metabolism dysregulation to sustained muscle injury. Defective mitophagy, mitochondrial DNA (mtDNA) depletion, and excessive reactive oxygen species (ROS) production create a self-amplifying loop with interferon-driven inflammation, whereas abnormal glycolysis, impaired fatty acid oxidation, and dysregulated tryptophan–kynurenine metabolism further shape the immunometabolic landscape of IIMs. These metabolic shifts not only contribute to muscle weakness and tissue degeneration but are also correlated with disease severity, autoantibody profiles, and treatment resistance. Emerging therapeutic strategies, including antioxidant approaches, mitochondrion-targeted agents, metabolic modulators, and exercise-based interventions, underscore the translational potential of targeting energy homeostasis. This review synthesizes current evidence on energy metabolism abnormalities in IIMs, integrates molecular findings with clinical implications, and highlights future directions for immunometabolic-based precision therapies.

## 1 Introduction

Idiopathic inflammatory myopathies (IIMs) represent a heterogeneous group of autoimmune disorders characterized by chronic muscle inflammation, progressive weakness, and systemic complications involving the skin, lungs, heart, and gastrointestinal tract (1). The major subtypes of IIMs, including dermatomyositis (DM), inclusion body myositis (IBM), antisynthetase syndrome (ASyS), immune-mediated necrotizing myopathy (IMNM), polymyositis (PM), and overlap myositis (OM), exhibit distinct histopathological features and immunopathogenic mechanisms (2). The global incidence of IIMs ranges from 0.2 to 2 per 100,000 person-years, with a prevalence ranging from 2 to 25 per 100,000 people, depending on the geographical region and population studied (3). The aetiology of IIM is primarily multifactorial, arising from a complex interplay among genetic susceptibilities (such as HLA haplotypes), epigenetic modifications (including DNA methylation and noncoding RNA regulation), environmental triggers (such as ultraviolet radiation, smoking, and viral infections), and socioeconomic determinants (4–6). Despite ongoing research efforts, the exact pathogenesis and progression of IIMs have not been fully clarified.

Immune inflammatory cell infiltration is traditionally regarded as the primary driver of myositis-induced weakness (6, 7). However, increasing evidence indicates that muscle dysfunction represents an important pathological component beyond inflammation. Multiple studies have reported that the severity of muscle weakness does not parallel the extent of immune cell infiltration. Weakness may even precede the appearance of inflammatory changes and often persists after their resolution (8–10). These observations suggest that mechanisms other than immune-mediated inflammation may contribute to the development or persistence of muscle weakness in myositis. Recent genetic evidence suggests that IIMs may involve not only immune dysregulation but also metabolic dysfunction. Novel susceptibility loci, such as NR1H4 and ABCB11, identified by Zhu et al., are functionally linked to bile acid levels, lipid metabolism and mitochondrial homeostasis, indicating that energy metabolism abnormalities may contribute to IIM pathogenesis (11–13). Indeed, various metabolic myopathies exhibit well-defined abnormalities in energy metabolism, such as mitochondrial myopathies characterized by defective oxidative phosphorylation (14), glycogen storage diseases involving abnormal glucose metabolism (15), and lipid storage myopathies resulting from fatty acid oxidation defects (11). These diseases all involve muscle weakness, highlighting the close association between energy metabolism and muscle function.

Emerging evidence supports this view, showing that abnormal energy metabolism contributes to the pathogenesis and progression of IIMs. Recent metabolomic and transcriptomic studies have identified energy metabolism as a vital player in IIM pathology, contributing to sustained muscle damage and immune dysregulation (16–18). In IIM patients, mitochondrial abnormalities, including mitochondrial DNA (mtDNA) deletions, impaired oxidative phosphorylation, and excessive production of reactive oxygen species (ROS), are consistently observed (19). Additionally, shifts in glucose metabolism towards glycolysis and dysregulation of the tricarboxylic acid (TCA) cycle are being increasingly recognized (20, 21). Furthermore, disruptions in lipid metabolism, including defects in fatty acid oxidation and lipid peroxidation, are linked to inflammation and muscle weakness (22). Amino acid metabolism, particularly dysregulation of the tryptophan-kynurenine (Trp-Kyn) pathway, is also involved in immune tolerance mechanisms through the suppression of M1 macrophages and CD4+T helper 1 (Th1) cells and the expansion of regulatory T cells (Tregs), with potential implications for disease progression and therapeutic intervention (23). The muscle microenvironment represents the critical interface where metabolic dysfunction and inflammation intersect. Within this space, infiltrating immune cells (e.g., macrophages and T cells) interact with myofibers, perpetuating a vicious cycle of mitochondrial dysfunction, oxidative stress, and metabolic reprogramming that ultimately drives myofiber energy depletion and persistent muscle weakness. Despite its importance, detailed insights into this complex crosstalk remain limited, and a comprehensive spatial and cellular understanding is still evolving.

In this review, we present a comprehensive synthesis of current knowledge on energy metabolism abnormalities in IIMs, with a focus on mitochondrial dysfunction, oxidative stress, and alterations in glucose, lipid, and amino acid metabolism. We further discuss their implications for disease pathogenesis, potential metabolic biomarkers, and novel therapeutic strategies aimed at restoring energy homeostasis in affected muscle tissues. By integrating insights from molecular mechanisms and clinical perspectives, this review aims to advance our understanding of metabolic energy dysfunction in IIMs and guide future research towards precision medicine approaches in IIM management.

## 2 Energy metabolism abnormalities in patients with IIMs

### 2.1 Mitochondrial dysfunction and oxidative stress

**Figure 1:**
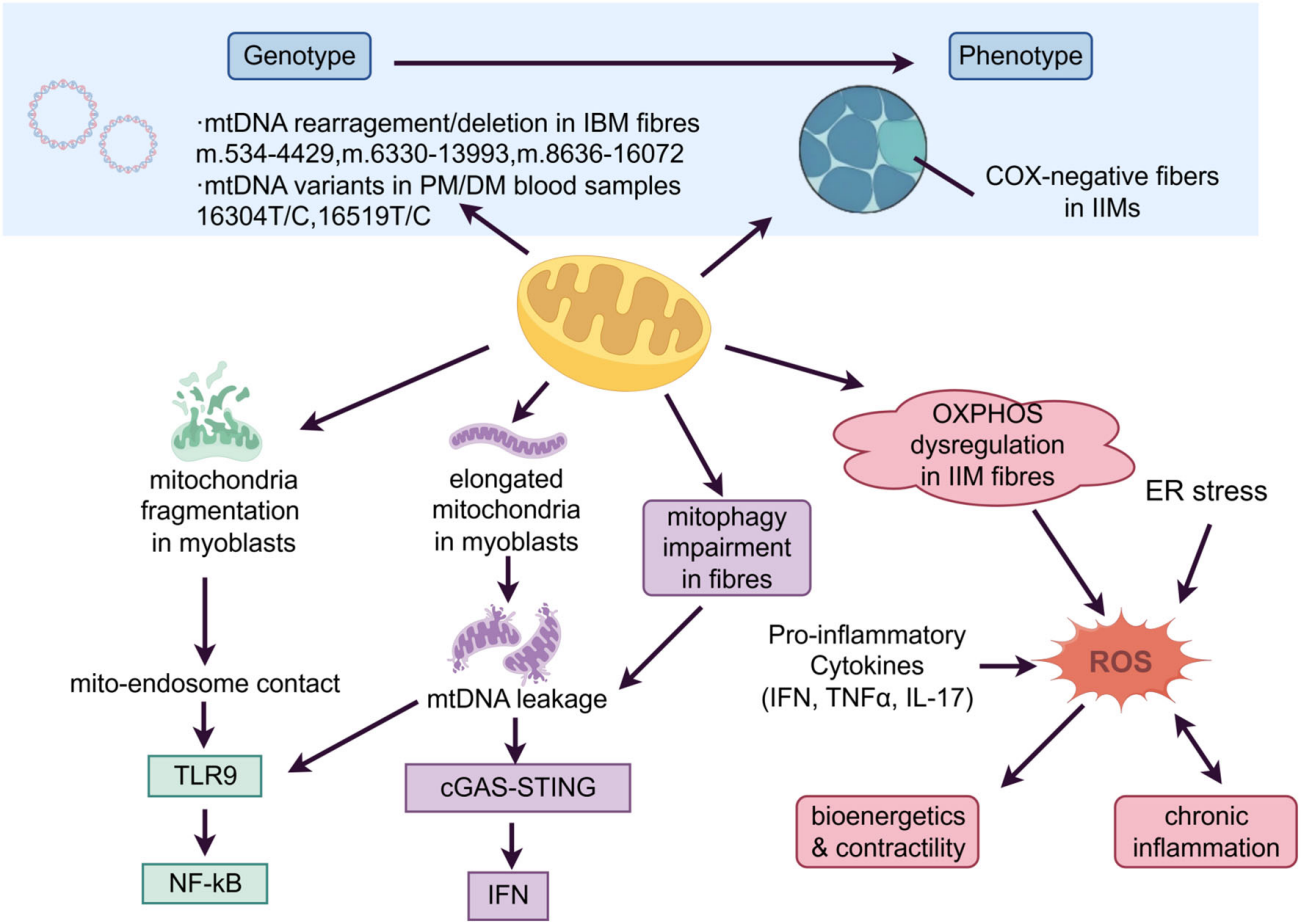
Mitochondrial dysfunction and oxidative stress in idiopathic inflammatory myopathies (IIMs). Genetic basis: mitochondrial DNA (mtDNA) depletion or deletions and D-loop variations lead to the loss of cytochrome c oxidase (COX). Immuno-metabolic coupling: impaired mitophagy, elongated and fragmented mitochondria lead to the activation of cyclic GMP-AMP synthase (cGAS)-stimulator of interferon genes (STING) and Toll-like receptor 9 (TLR9)–nuclear factor-kappa B (NF-κB) cascades. Reactive oxygen species (ROS) production: endoplasmic reticulum (ER) stress and pro-inflammatory cytokines promote the excessive production of ROS, which in turn sustains chronic inflammation and compromises bioenergetics and contractility.

As the central organelle for cellular energy production, mitochondria are crucial for maintaining metabolic homeostasis and muscle integrity (24). Multiple lines of evidence have established that mitochondrial dysfunction and oxidative stress are essential contributors to IIM pathogenesis, which links cellular energy failure to inflammation and tissue degeneration (as shown in **Figure 1**).

#### 2.1.1 Genetic basis of mitochondrial abnormalities

Genetic investigations first highlighted mitochondrial abnormalities. In the IBM, analyses of muscle biopsy specimens revealed that complex rearrangements and heteroplasmic deletions tend to cluster within three major regions (m.534–4429, m.6330–13993, and m.8636–16072) (25, 26). In contrast, PM/DM is characterized by the preferential accumulation of mtDNA point variants in the displacement loop (D-loop), particularly at 16304T/C and 16519T/C, identified from peripheral blood samples. The 16519C allele is significantly associated with antinuclear antibody (ANA) positivity and elevated IL-2 production, whereas 16304C is correlated with reduced IL-4 levels. These correlations indicate that mtDNA variants may contribute to immune dysregulation in patients with PM/DM (27).

#### 2.1.2 Integrative omics insights into mitochondrial dysfunction

Omics-level approaches provide consistent and multifaceted evidence for mitochondrial involvement across IIM subsets while also revealing disease-specific nuances. At the transcriptomic level, Zhong et al. identified 20 nuclear-encoded hub genes through weighted gene coexpression network analysis (WGCNA) across muscle biopsies from 13 muscle disease groups. They revealed a coordinated pattern characterized by upregulation of interferon (IFN)-stimulated genes alongside suppressed key subunits of mitochondrial oxidative phosphorylation (OXPHOS), including cytochrome c oxidase (COX), NDUF and ATP synthase. This linked IFN signalling to impaired energy metabolism in juvenile dermatomyositis (JDM) (28). Qin et al. performed a transcriptomic analysis of muscle biopsies from PM patients, and their results revealed downregulation of OXPHOS and activation of type I IFN signalling, which suggested impaired mitochondrial metabolism in PM. However, this study was primarily based on bioinformatic re-analysis of publicly available GEO datasets, for which clinical annotation was limited and heterogeneity across datasets may exist (29). In this study, reduced OXPHOS signatures may reflect interferon-associated metabolic suppression accompanied by mitochondrial defect. In IBM, IFNγ–associated signalling was enriched in attacked myofibers and correlated with the presence of adjacent CD8⁺ T cells (30). In parallel, Kleefeld performed proteomic analysis of IBM muscle. Their results showed increased antigen presentation and T cell–mediated immune pathways, accompanied by the depletion of mitochondrial respiratory chain and OXPHOS components (NADH dehydrogenase 2) (31). This alteration may reflect mitochondrial dysfunction associated with chronic immune activation and a sustained oxidative stress environment. In contrast, bulk RNA sequencing of muscular specimens by Izuka et al. revealed a distinct enrichment of OXPHOS and mitochondrial respiration–related gene signatures in anti–aminoacyl-tRNA synthetase antibody–positive dermatomyositis (ARS-DM). These gene modules were found to be positively correlated with serum creatine kinase (CK) levels. The authors also reported correlations between CK levels and infiltrating monocytes and myeloid dendritic cells (32). These observations reflect a metabolic adaptation of immune cells (especially monocytes) to persistently reside in muscle tissue and execute effector functions. Additionally, Hamann et al. performed transcriptional profiling to identify the differential expression of long noncoding RNAs in muscle biopsies from patients with Jo-1 myositis and IBM, and they reported that OXPHOS and mitochondrial dysfunction were the most enriched pathways in both diseases (33). Such enrichment may coexist with structural mitochondrial abnormalities in chronically inflamed muscle, which reflect mitochondrial dysfunction or metabolic remodeling in muscle cells themselves due to chronic inflammation. Taken together, the directionality of OXPHOS alterations varies across studies, representing a complex, multicellular metabolic state in myositis. In addition, integrated methylation and transcriptomic profiling revealed the upregulation of the expression of harakiri (HRK), a mitochondrion-localized pro-apoptotic factor, in the skeletal muscle of patients with PM/DM. The upregulation of HRK expression was found to be associated with reduced mitochondrial membrane potential and impaired regenerative capacity of skeletal muscle (34).

Spatial transcriptomic studies have added a crucial histological context, particularly in JDM. These investigations demonstrated that mitochondrial dysregulation is a consistent and fundamental pathological feature. For example, Syntakas et al. reported downregulated respiratory chain pathways in JDM muscle irrespective of IFN activity or muscle strength (35). Tragin et al. performed muscle spatial transcriptomic on JDM and further reported a loss of the normal mitochondrial signature, which was replaced by pathological programs that persisted even after clinical remission, suggesting that mitochondrial abnormalities may outlast overt inflammation (36). These findings collectively highlight mitochondrial dysfunction as a potential therapeutic target independent of the inflammatory cascade.

Complementing transcriptomic data, proteomic and single-cell studies have refined our understanding at the protein and cellular level. Peterson et al. identified 13 dysregulated mitochondria inner-membrane proteins in the muscle of IIMs, including specific subunits of Complex I (NDUFB3, NDUFB5, NDUFB6, MT-ND2) and Complex IV (COX7C, COX7A2L, COX6A2), linking structural defects to impaired ATP synthesis (37). Additionally, Ding et al. conducted single-cell transcriptomic profiling of peripheral blood mononuclear cells (PBMCs) obtained from patients with ASyS. They revealed a unique immunometabolic landscape featuring coordinated activation of OXPHOS and IFNγ–TNF signalling in monocytes and NKT cells, along with MAIT cell loss and FOXM1–IRF1-mediated transcriptional remodelling (38).

#### 2.1.3 Phenotypic and functional manifestations of mitochondrial dysfunction

Histological, ultrastructural, and *in vivo* functional analyses provide convergent evidence for mitochondrial dysfunction as a core feature of IIMs. A key morphological hallmark is the presence of COX-deficient fibres. This was initially highlighted in a study of 30 patients (15 DM, 12 PM, 3 IBM), which reported a significantly higher percentage of such fibres than controls did, with a notable perifascicular pattern in DM that coincided with capillary loss (39). This abnormality extended to other IIM subsets, including a higher frequency of anti-3-hydroxy-3-methylglutaryl-CoA reductase (HMGCR) IMNM (40) and the presence of scattered COX-deficient fibres in ASyS, although the characteristic perifascicular pattern remained distinct from that of DM (41).

Beyond COX deficiency, studies have revealed a broader spectrum of mitochondrial abnormalities. In patients with IBM and a group termed polymyositis with mitochondrial pathology (PM-Mito), multiple mitochondrial abnormalities were shown in skeletal muscle biopsy specimens (43). These include disorganized cristae, reduced mtDNA copy number with multiple deletions, and dysregulation of inner mitochondrial membrane proteins. PM-Mito, which is defined histopathologically by endomysial inflammation, invasion of non-necrotic fibres, and >3% COX-deficient fibres in skeletal muscle biopsies (42), has therefore been proposed as a precursor stage within the IBM spectrum. Notably, marked mitochondrial abnormalities were already evident at this early stage and preceded tissue remodelling as well as infiltration by specific T-cell subpopulations (43). This indicates that mitochondrial dysfunction represents an early pathogenic event in IBM. Multiple defects, including reduced mitochondrial genome content, diminished membrane potential, and impaired mitochondrial elongation, have been confirmed in the fibroblasts of patients with IBM (44). Furthermore, studies in sporadic IBM (sIBM) patients have demonstrated decreased COX activity relative to that of citrate synthase in both skeletal muscle and PBMCs, together with impaired mitochondrial dynamics in skeletal muscle (45).

These structural abnormalities are directly linked to impaired energetics *in vivo*. Phosphorus magnetic resonance spectroscopy (³¹P-MRS) studies on patients with DM and PM have revealed reduced ATP production, impaired proton efflux, and markedly prolonged phosphocreatine recovery after exercise, indicating a severe deficit in mitochondrial energy regeneration. Interestingly, these metabolic defects have been attributed primarily to impaired capillary perfusion rather than an intrinsic respiratory chain defect, suggesting a convergence of vascular and metabolic pathology in limiting ATP generation in IIMs (46).

At the clinical level, mitochondrial abnormalities were found to be associated with disease refractoriness and poor prognosis across IIM subtypes. In a retrospective analysis of 25 patients, Lauletta et al. reported that patients with non-IBM myositis with mitochondrial abnormalities—particularly PM-Mito—had a greater proportion of COX-negative fibres. This feature was associated with treatment refractoriness and poor outcomes, suggesting that mitochondrial dysfunction may serve as a prognostic marker (47). In support of this association across subtypes, Turnier et al. identified a refractory JDM subgroup characterized by upregulation of mitochondrial dysfunction-related genes in skin samples (48), and Lauletta et al. described a case of anti-Mi-2 DM with diffuse mitochondrial abnormalities at muscle biopsy that exhibited poor therapeutic response (49). Moreover, Notarnicola et al. identified autoantibodies against a mitochondrial complex I subunit in plasma samples collected from IBM patients, revealing a new autoimmune target that might be specifically associated with IBM (50).

In conclusion, accumulating evidence positions mitochondrial dysfunction as a central pathological mechanism in IIMs, influencing not only muscle structure and energetics but also treatment refractoriness and disease progression. A comprehensive understanding of these mechanisms is vital for advancing the development of targeted therapeutic strategies.

#### 2.1.4 Mechanistic implications of mitochondrial dysfunction

Phenotypic evidence of mitochondrial damage prompted the question of its underlying mechanism. Recent work has begun to elucidate these links. For instance, perifascicular COX deficiency in DM was shown to result from mtDNA depletion, leading to a selective loss of mtDNA-encoded respiratory chain complexes I and IV, while the nuclear-DNA-encoded complex II remained intact. This finding established a direct structural mechanism linking mtDNA damage to respiratory chain failure (51).

Moreover, the immunometabolic context of these mitochondrial defects is being increasingly defined. COX-deficient fibres in DM are frequently associated with upregulated expression of perifascicular major histocompatibility complex class I (MHC-I), whereas in IBM, they are accompanied by significant T-cell and macrophage infiltration, positioning mitochondrial pathology within a prominent inflammatory microenvironment (51, 52). Thoma et al. demonstrated in an *in vitro* model of human skeletal muscle that MHC-I overexpression directly impaired mitochondrial function, characterized by decreased respiration and increased proton leakage. This effect was synergistically exacerbated by type I interferon, highlighting a direct pathway through which key immune signals in myositis drive mitochondrial injury (53). A key unresolved question concerns the causal directionality between mitochondrial failure and inflammation. It remains unclear whether one is the primary driver or whether both operate within a self-reinforcing cycle. The detailed mechanisms—including oxidative stress and defective mitophagy, which are often accompanied and potentiated by innate immune activation—are believed to constitute this vicious cycle.

##### 2.1.4.1 Defective mitophagy

Defective mitophagy, the impaired selective autophagy of mitochondria, is involved in the pathogenesis of inflammatory myopathies through failure to clear damaged organelles, which subsequently disrupts bioenergetics and triggers inflammation. Evidence for this defect varies across IIM subsets. In IBM, immunohistochemical studies of muscle biopsies revealed impaired degradation of autophagic flux marked by increased LC3 and p62 accumulation compared with that in PM (54). Subsequent studies revealed elevated expression of BNIP3, a Bcl-2 family protein that initiates mitophagy, at both the mRNA and protein levels in muscle biopsies from sIBM patients, thus providing further evidence of impaired mitochondrial mitophagy in the pathogenesis of this disease (55). Further support in the IBM muscle included increased levels of the mitophagy marker phosphorylated ubiquitin (p-S65-Ub), which correlated with muscle weakness (56); initiation-deficient autophagic flux, as evidenced by reduced LC3B-II and p62 accumulation; and diminished autophagosome formation in patient-derived fibroblasts (44). Transcriptomic analysis of induced pluripotent stem cell (iPSC)-derived myotubes from IBM patients further revealed downregulation of mitochondrial and autophagy-related genes, reinforcing the link to energy failure (57). In IMNM, pathological accumulation of degenerated mitochondria in skeletal muscle similarly suggested a failure of mitochondrial quality control (58).

The accumulation of damaged mitochondria due to impaired mitophagy leads to the cytosolic leakage of mtDNA. This mtDNA acts as a damage-associated molecular pattern (DAMP) that activates innate immune signalling. In IBM muscle biopsies, mitochondrial damage and cytosolic mtDNA escape are associated with an early immune response driven by activation of the cyclic GMP-AMP synthase (cGAS)-stimulator of interferon genes (STING) pathway (43). Consistently, muscle biopsies from IIM patients showed increased expression of cGAS, STING, and related molecules (59). The causal link was further strengthened by experimental models such as Fis1 or Drp1 knockdown. In these models, disruption of mitochondrial dynamics induced mitochondrial elongation and cytosolic mtDNA leakage. This subsequently activated the cGAS–type I IFN pathway and promoted muscle inflammation (60). Notably, Irazoki et al. demonstrated that disturbed mitochondrial dynamics was a potent trigger of inflammation: in myoblasts, mitochondrial fragmentation activated Toll-like receptor 9 (TLR9)–nuclear factor-kappa B (NF-κB) signalling, whereas mitochondrial elongation induced both the NF-κB and the cGAS/type I IFN pathways via mislocalized mtDNA. Consistently, mice with muscle-specific deletion of mitofusin-1 developed TLR9-driven inflammation and muscle atrophy, which were ameliorated by anti-inflammatory treatment (60). This supports a model in which various forms of mitochondrial damage can initiate inflammatory cascades in addition to defective mitophagy.

Impaired mitophagy in IIMs creates a vicious cycle: it not only undermines cellular bioenergetics by retaining dysfunctional mitochondria but also promotes chronic inflammation through mtDNA-mediated activation of the cGAS-STING pathway, thus bridging metabolic dysfunction and innate immune activation.

##### 2.1.4.2 Oxidative stress and ROS

Oxidative stress is established as a central pathogenic mechanism in IIMs, arising from mitochondrial dysfunction and operating within a self-sustaining vicious cycle with inflammation. At a fundamental level, endoplasmic reticulum (ER) stress acts as a key initiator, where ER stress-induced Ca²⁺ release and its subsequent mitochondrial uptake lead to increased membrane potential and ROS production (61). This core mechanism is complemented by different triggers. For instance, in JDM, IFN-α-induced inflammation promoted mitochondrial calcification in skeletal muscle fibres via ROS, whereas in parallel, altered metabolism in CD14⁺ monocytes led to the release of oxidized mtDNA, which activated interferon pathways and linked mitochondrial stress to systemic autoimmunity (62, 63). Notably, mitochondrial gene expression was inversely associated with the type I IFN score in CD14^+^ monocytes of pretreatment JDM (63). Similarly, in a model of IFNγ-driven myositis, IFNγ induced ROS overproduction, mitochondrial damage, and OXPHOS suppression. At the clinical level, biopsies from DM patients displayed an inverse correlation between IFNγ signatures and mitochondrial gene expression. It is worth noting that metabolic alterations were detectable before overt immune cell infiltration, and preventive ROS buffering attenuated subsequent inflammation (16). This provided strong experimental evidence that mitochondrial dysfunction and oxidative stress may causally contribute to the amplification of inflammatory cascades. Additionally, IFN-β impaired mitochondrial function in DM, and IL-17 and TNFα synergistically exacerbated ER stress and mitochondrial ROS in IIM myoblasts (64). Meyer et al. conducted mechanistic experiments and demonstrated that IFN-β induces mitochondrial dysfunction through ROS in DM. In parallel, mitochondrial respiratory capacity was inversely correlated with IFN signature (65). Moreover, Zhang et al. established an IMNM mouse model using patient-derived anti-signal recognition particle (anti-SRP) immunoglobulin G. They demonstrated that anti-SRP antibodies induced cardiac diastolic dysfunction through ROS overproduction and mitochondrial injury (66).

The pathological effects of ROS are executed through two major pathways. First, ROS directly impair muscle bioenergetics by uncoupling the electron transport chain, reducing ATP synthesis, and causing an energy deficit while also compromising contractility through oxidative modification of myofibrillar proteins, leading to fatigue (67, 68). Second, ROS act as potent immunomodulators, activating pathways such as NF-κB to sustain proinflammatory cytokine expression, which in turn feeds back to amplify the initial triggers (e.g., ER stress), creating a bidirectional reinforcement loop (69).

The evidence that the damage cycle of ROS can be interrupted confirms its significant role in IIM pathology. Interventions such as blocking IFNγ or JAK signalling or buffering ROS with N-acetylcysteine (NAC) have been shown to restore mitochondrial integrity, improve OXPHOS, and reduce inflammation in various models, including human myoblasts and the anti-SRP IgG model (16, 65, 66). Together, these data indicate that self-maintaining crosstalk between oxidative stress and inflammation is a key mechanism underlying the persistent myofibril injury observed across the spectrum of IIMs.

### 2.2 Glucose metabolism

**Figure 2:**
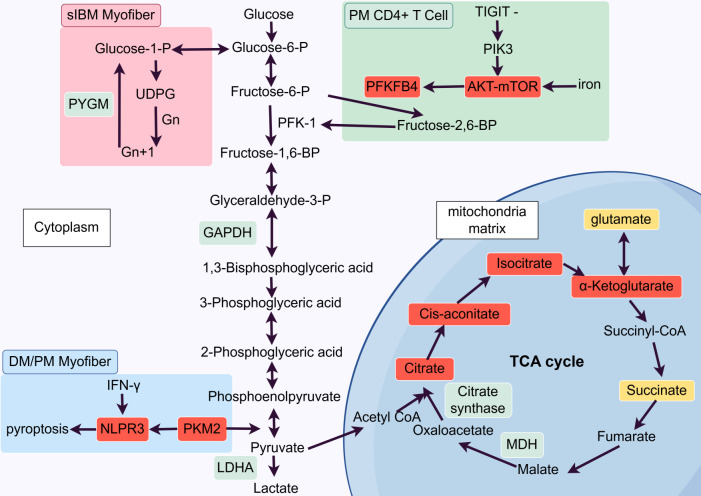
Glucose metabolic reprogramming across IIM subsets. Glycolytic flux: dermatomyositis (DM)/polymyositis (PM) muscles exhibit pyruvate kinase M2 (PKM2) over-expression, which promotes glycolysis and activates the NLRP3 inflammasome to induce myofiber pyroptosis; conversely, sporadic inclusion body myositis (sIBM) shows down-regulation of GAPDH, lactate dehydrogenase A (LDHA) and glycogen phosphorylase (PYGM). Tricarboxylic acid (TCA) cycle heterogeneity: PM biopsies display reduced activity of citrate synthase and malate dehydrogenase (MDH); inclusion body myositis (IBM) specimens display variable results across studies, including reports of reduced succinate and glutamate levels as well as elevated levels of succinate, glutamate, citrate, cis-aconitate, isocitrate, and α-ketoglutarate. Immunometabolic coupling: iron–AKT–mammalian target of rapamycin (mTOR) signalling up-regulates the expression of the 6-phosphofructo-2-kinase/fructose-2,6-bisphosphatase 4 gene (*PFKFB4*) in CD4⁺ T cells from patients with PM; T-cell immunoreceptor with Ig and ITIM domains (TIGIT)-deficient autoreactive T cells amplify glycolysis through PI3K/AKT/mTOR pathway.

Glucose metabolism is important for both muscle function and immune regulation. It provides energy to support muscle activity and immune activation. In IIMs, this finely tuned system becomes dysregulated (as shown in **Figure 2**), which leads to metabolic reprogramming in both myofibres and immune cells that ultimately fuels inflammation and tissue damage.

Recent studies have identified multiple glycolytic abnormalities in IIMs. For example, Liu et al. reported elevated glycolytic flux in the muscles of DM and PM patients, which correlated with the upregulation of pyruvate kinase M2 (PKM2), a rate-limiting enzyme in glycolysis. Moreover, the authors reported that a PKM2 inhibitor blocked IFNγ-induced activation of the NLRP3 inflammasome and pyroptosis in myotubes. These findings suggest that upregulated glycolysis may activate the NLRP3 inflammasome and trigger pyroptotic cell death in muscle tissue. Thus, glycolysis not only fuelled energy demand, but is functionally required for NLRP3 activation and pyroptosis (20). This supports a metabolism-to-inflammation axis in DM and PM. Additionally, overexpression of PKM2 and mitochondrial ATPase inhibitor factor 1 (IF1) in DM muscle was found to act synergistically to promote a glycolytic phenotype and partially suppress glucose oxidation. This gave rise to a microenvironment enriched in oncometabolites that may favour neoplasia. Importantly, the authors identified the glycolysis-promoting proteins PKM2 and IF1 as specific biomarkers of DM, providing both diagnostic value and mechanistic insight into the biochemical link between this IM and oncogenesis (70). In contrast to DM/PM, sIBM exhibited distinct glycolytic alterations. Santacatterina et al. reported significant downregulation of key glycolytic enzymes, including glyceraldehyde-3-phosphate dehydrogenase (GAPDH) and lactate dehydrogenase A (LDHA), in muscles from sIBM patients. This metabolic divergence was further supported by the reduced expression of two key enzymes: glycerol-3-phosphate dehydrogenase 1 (GPD1), which is essential for mitochondrial NADH shuttle systems, and glycogen phosphorylase (PYGM), the gatekeeper enzyme of glycogenolysis (a pathway upstream of glycolysis) (70). These divergent glycolytic patterns may reflect distinct pathogenic contexts. In DM, enhanced glycolysis appears to be inflammation-driven and functionally linked to NLRP3 activation and pyroptosis. In contrast, the coordinated downregulation of multiple glycolytic enzymes in sIBM suggests reduced glycolytic flux, impaired NADH shuttle activity, and limited glycogen mobilization. This points to impaired energy production associated with degenerative muscle pathology rather than adaptive metabolic remodeling.

Beyond changes in glycolysis, functional changes in the TCA cycle have been observed, although the findings may vary by disease model and stage. Sunitha et al. reported that several enzymes of the TCA cycle were downregulated in muscle tissues from patients with PM. They reported impaired activity of citrate synthase (CS) and malate dehydrogenase (MDH) (21). Another study by Boncompagni et al. revealed that the levels of intermediates and byproducts of the TCA cycle, such as succinate and glutamate, were significantly reduced in the skeletal muscles of muscle creatine kinase–β-amyloid precursor protein (MCK-βAPP) transgenic mice, which exhibit myopathic features of IBM (71). In contrast, Naddaf et al. reported elevated TCA cycle intermediates (succinate, fumarate, citrate, cis-aconitate, isocitrate, and α-ketoglutarate) and anaplerotic amino acids (e.g., glutamate, aspartate, asparagine), alongside reduced proximal glycolytic metabolites and increased pentose phosphate pathway (PPP) activity in IBM muscle samples. These results reflected metabolic rerouting from glycolysis towards the TCA cycle and PPP (56). The seemingly opposite conclusions between Boncompagni et al. and Naddaf et al. could reflect subtype-specific disease mechanisms. In PM, acute inflammation may suppress mitochondrial enzyme expression and activity, leading to impaired TCA capacity. IBM could show chronic, mitochondria-centred dysfunction with impaired mitophagy. In this context, TCA enzymes may be relatively preserved while metabolic flux is disrupted, leading to the accumulation of intermediates. Further research is needed to reconcile these findings.

The impact of glucose metabolic reprogramming on immune cell function has also been increasingly recognized as a key driver of inflammation. By integrating single-cell RNA sequencing, flow cytometry, and multiplex immunohistochemistry, Ye et al. comprehensively characterized the adaptive immune landscape of peripheral B and T cells and affected lung tissue in MDA5+ DM. Their analyses revealed pronounced type I IFN activation accompanied by metabolic reprogramming of immune cells. OXPHOS, pyruvate metabolism, and glycolysis were markedly enriched in peripheral B and T cells (72). Additionally, Lai et al. performed RNA-seq analysis in CD4⁺ T cells from IIM patients and reported that iron promoted glucose metabolism by upregulating the expression of the 6-phosphofructo-2-kinase/fructose-2,6-bisphosphatase 4 gene (*PFKFB4*) through the AKT–mammalian target of rapamycin (mTOR) signalling pathway. This metabolic shift subsequently directed CD4⁺ T cells towards a proinflammatory phenotype (73). Another study in the PM reported reduced expression of T-cell immunoreceptor with Ig and ITIM domains (TIGIT), an inhibitory receptor that normally contributes to immune tolerance by restraining T–cell-mediated autoimmunity. The authors demonstrated that TIGIT-deficient autoreactive CD4⁺ T cells exhibited enhanced glycolysis through enhanced CD28-mediated PI3K/AKT/mTOR co-stimulatory pathway, which not only promoted their survival and effector functions but also exacerbated muscle inflammation (74).

### 2.3 Lipid metabolism

Emerging lipidomic investigations have revealed profound perturbations in lipid homeostasis as vital mediators of the immunometabolic axis in IIMs (Lipid metabolism abnormalities in IIMs are summarized in **Table 1**). These metabolic disturbances act through multiple pathological mechanisms, driving both myofibril degeneration and systemic disease manifestations (75).

**Table 1:** Lipid metabolism abnormalities in IIMs.

Dimension	Key findings	IIM subtypes	Clinical/mechanistic implications
Systemic Lipid Profiles	↑ Triglycerides, ↓ HDL-C; altered PC/TG species; increased palmitic acid	PM/DM	Reflect inflammatory lipid dysregulation; correlated with CK and muscle strength; partly reversible with treatment (75–77)
Antibody-Specific Lipid Signatures	Anti-MDA5: sphingolipid pathway activation (ILD/fibrosis); Anti-Jo-1: lysophospholipid accumulation	DM	Indicate disease activity and organ involvement; useful for risk stratification (78)
Muscle Cell Lipid Metabolism	Reduced palmitate oxidation; ↓ incomplete fatty acid oxidation; ↓ diglyceride synthesis	IIM overall	Impaired oxidative and non-oxidative lipid pathways; reversible after exercise training (22)
Juvenile DM Lipid Abnormalities	↑ long-chain ACs; ↑ several ceramides	JDM	Reflect impaired mitochondrial β-oxidation (79)
IBM Lipid Disturbances	Free-cholesterol accumulation; ↑ LDLR/VLDLR/LRP; AC abnormalities (sex-dependent)	IBM	Indicate disordered cholesterol handling; certain AC species inversely correlated with disease duration (56, 80)
Immunometabolic Lipid Changes	DEPTOR–mTOR may regulate lipid metabolism in IIMs; ↑ palmitoleic acid in PM; dysfunctional HDL with ↓ antioxidant capacity in IIMs	PM/IIM overall	Link lipid dysregulation to immune imbalance and vascular inflammation; related to disease activity (81–83)

IIM, idiopathic inflammatory myopathy; HDL-C, high density lipoprotein cholesterol; PC, phosphatidylcholine; TG, triacylglycerol; PM, polymyositis; DM, dermatomyositis; CK, creatine kinase; ILD, interstitial lung disease; AC, acylcarnitine; JDM, juvenile dermatomyositis; IBM, inclusion body myositis; HDL, high-density lipoprotein.

Accumulating evidence from serological and metabolic studies has supported a consistent pattern of defective lipid handling in IIMs. At the serological level, a pro-atherogenic lipid profile characterized by elevated triglycerides and reduced high density lipoprotein cholesterol (HDL-C) is consistently observed in newly diagnosed or untreated PM/DM patients across independent cohorts (76, 77). Alterations in the levels of phosphatidylcholine (PC) and triacylglycerol (TG) species, along with an increased proportion of palmitic acid, are further characterized by targeted lipidomics in PM/DM. Moreover, the levels of several lipid species are correlated with CK levels and muscle performance. Notably, abnormalities in lipid metabolism can be partially normalized following immunosuppressive therapy (75).

In further detail, lipid disturbances displayed distinct patterns across disease subtypes. In patients with DM, disturbed glycerophospholipid and fatty acid metabolism displayed antibody-specific patterns. Activation of the sphingolipid pathway in anti-MDA5^+^ DM was associated with interstitial lung disease (ILD) and fibrosis, whereas lysophospholipid accumulation in anti-Jo-1^+^ DM correlated with muscle and joint involvement. Importantly, these antibody-dependent shifts parallel disease activity and lung injury severity (78). In terms of muscle alterations, Nemec et al. treated primary muscle cells derived from IIM patients with palmitate, and they identified that both oxidative and non-oxidative lipid metabolism were reduced and adipose triglyceride lipase (ATGL) levels were elevated. These changes indicated concurrent impairments in both oxidative and non-oxidative lipid pathways. Moreover, the authors also reported aberrant OXPHOS complex IV/V regulation and reduced AMP-activated protein kinase (AMPK) activation. Notably, these abnormalities were ameliorated by 6 months of training (22). Additionally, in JDM, Dvergsten et al. reported that the levels of 12 individual acylcarnitines (ACs), which were mostly long chain, and 3 ceramides, were significantly greater. This was interpreted to reflect dysregulated mitochondrial fatty acid β-oxidation (79). In the IBM, vacuolated fibres display free cholesterol accumulation together with elevated expression of lipoprotein receptors such as LDLR, VLDLR, and LRP, indicating dysregulated intramyofibre cholesterol handling (80). Moreover, multiple AC alterations were identified in the muscle metabolomic profile of IBM, which exhibited sex-dependent patterns. Additionally, the levels of certain acylcarnitine species were inversely correlated with disease duration (56).

Notably, the link between abnormal lipid metabolism and inflammation has gradually drawn attention. Experimental and clinical studies are beginning to elucidate the underlying mechanisms. Xie et al. demonstrated the important role of DEPTOR–mTOR signalling in regulating lipid metabolism and inflammatory homeostasis in PBMCs from healthy donors. They suggested that dysregulation of this pathway may be relevant to autoimmune diseases, including IIMs (81). Subsequent studies by Yin et al. revealed that increased palmitoleic acid in PBMCs is a biomarker of PM and that inhibition of mTORC1 activity could decrease palmitoleic acid levels (82). In addition, Bae et al. demonstrated that high-density lipoprotein (HDL) dysfunction in IIM patients resulted in reduced antioxidant capacity and increased proinflammatory activity, which contributed to vascular inflammation and endothelial injury. The authors reported that these changes were correlated with disease activity and plasma myeloperoxidase (MPO) levels and potentially linked lipid metabolism to the risk of cardiovascular events (83).

In summary, these findings underscore the close interplay between lipid metabolism and immune dysregulation. A deeper understanding of this interaction may shed light on novel mechanisms of IIMs.

### 2.4 Amino acid metabolism

**Figure 3:**
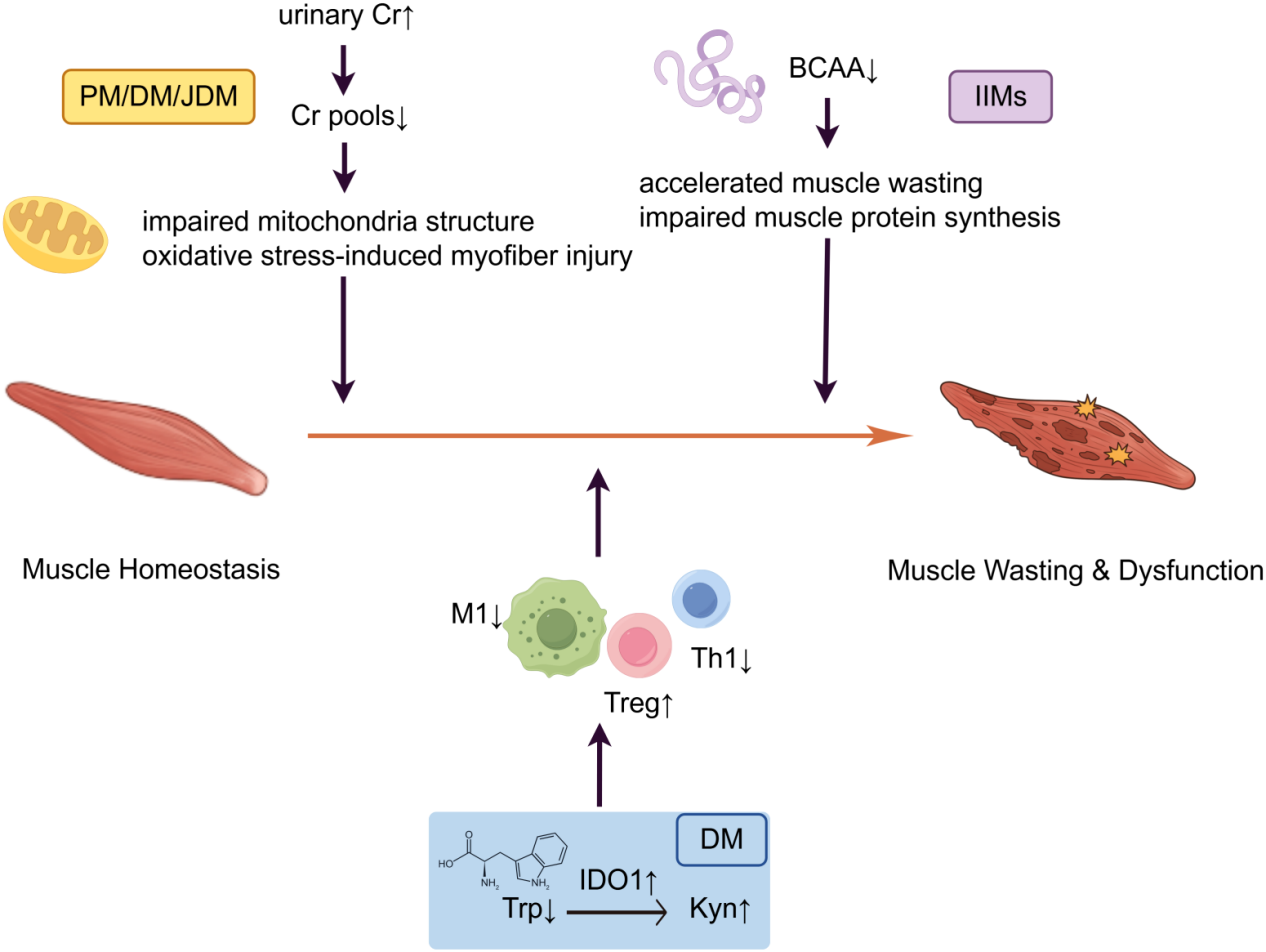
Amino acid metabolic dysregulation in IIMs. branched-chain amino acid (BCAA) depletion: Reduced serum levels of BCAA accelerate muscle wasting and impair muscle protein synthesis. Tryptophan (Trp)-kynurenine (Kyn) pathway: Significant upregulation of indoleamine 2,3-dioxygenase 1 (IDO1) drives Trp depletion and Kyn accumulation, which suppress M1 macrophages (M1) and CD4+T helper 1 (Th1) cells and expand regulatory T cells (Tregs). The loss of creatine (Cr) pools: Increased urinary Cr excretion and an elevated Cr/age-adjusted creatinine (Cn) ratio reflect the loss of Cr pools, which impairs mitochondria structure and reduces myofiber resistance to oxidative stress.

Amino acids are vital to muscle protein turnover and bioenergetic supply (84). Increasing evidence indicates that disruption of amino acid metabolism is an important component of energy imbalance in IIMs (Amino acid metabolic dysregulation in IIMs is summarized in **Figure 3**).

Systemic metabolomic analyses of serum and muscle tissue revealed widespread disturbances in amino acid profiles across IIM subtypes. Using NMR-based metabolomics, Guleria et al. found that serum levels of branched-chain amino acids (BCAAs)—particularly leucine and valine—were significantly lower in patients with active IIMs than in patients in remission. Given that BCAAs serve as essential substrates for maintaining muscle mass, this metabolic disturbance is correlated with impaired muscle protein synthesis and accelerated muscle wasting. Additionally, these BCAA level fluctuations may serve as potential biomarkers for distinguishing disease activity states (85). Similarly, Zhang et al. reported decreased phenylalanine and tryptophan levels in patients with DM. They highlighted disruptions in aminoacyl-tRNA biosynthesis and aromatic amino acid metabolism by pathway enrichment analysis, which supported the diagnostic and monitoring value of amino acid metabolic signatures in IIMs (86). Additionally, Naddaf et al. reported widespread amino acid alterations in IBM muscles, including elevated anaplerotic amino acids that replenished the TCA cycle. Among these, cysteine—a glutathione precursor—was shown to be positively correlated with muscle weakness, whereas carnosine and sarcosine were inversely correlated with strength. Such observations support the integral role of amino acid metabolic reprogramming in driving energy imbalance and muscle pathology in IIMs (56).

In addition to serum and muscle profiling, urinary metabolomics provided additional insights into ongoing muscle damage and metabolic alterations.Chung et al. reported that urinary levels of creatine (Cr), taurine, glycine, citrate, and choline-containing metabolites were significantly elevated in patients with DM/PM (87). In JDM, an increased urinary Cr/age-adjusted creatinine (Cn) ratio was found to positively correlate with cumulative disease damage, indicating that ongoing muscle injury drives Cr leakage and the depletion of intramuscular reserves (88). Importantly, experimental studies have demonstrated that Cr can protect mitochondrial structure and function and counteract oxidative stress-induced myofiber injury (89), which suggests that the loss of Cr pools may directly contribute to muscle weakness in IIMs.

At the immunometabolic level, the Trp-Kyn pathway emerged as a key nexus linking metabolism to immune regulation. This pathway was found to be persistently activated in patients with DM. Current evidence suggests that indoleamine 2,3-dioxygenase 1 (IDO1), an immunomodulatory enzyme that metabolizes tryptophan to kynurenine, is activated during tumorigenesis and promotes immune response evasion. In the muscle tissue of DM patients, a meta-analysis by Aljabban et al. revealed marked upregulation of IDO1 (90). Subsequent studies in 57 DM patients by Wu et al. confirmed enhanced Trp-Kyn metabolism, which was characterized by IDO1-mediated tryptophan depletion and kynurenine accumulation. Although kynurenine has immunosuppressive effects that could in theory limit autoimmunity, clinical studies have paradoxically linked its accumulation to more severe disease and poorer outcomes (23).

Overall, amino acid metabolic abnormalities represent a key feature of IIMs, which links defective muscle energetics with immune dysregulation and disease severity.

## 3 Clinical interventions for energy metabolism in idiopathic inflammatory myopathies

### 3.1 Pharmacological treatments

Current pharmacological management strategies for IIMs primarily target immune-mediated inflammation. Many patients experience treatment resistance or relapse, and long-term glucocorticoid exposure is associated with a substantial risk of adverse effects and metabolic complications. To sustain disease control while minimizing the steroid burden, immunosuppressive agents such as methotrexate, azathioprine, or mycophenolate mofetil are frequently combined (5). Some immunomodulatory therapies have been reported to influence cellular metabolism pathways. For instance, JAK inhibitors can reverse interferon-associated transcriptional programs involving mitochondrial and metabolic genes (91). IVIG can induce autophagy in immune cells, which is a process required for the suppression of inflammatory responses (92). In addition, B cell–targeted therapies deplete autoreactive B cells (93, 94), and therefore may reshape the immunometabolic environment. These observations suggest a potential link between immunomodulation and metabolic regulation. However, these regimens rarely achieve full functional recovery, indicating an urgent need for novel, mechanism-based therapies targeting metabolic and bioenergetic disturbances that represent potential therapeutic avenues in IIMs.

With mitochondrial dysfunction increasingly recognized as a central driver of energy dysregulation in IIMs, therapeutic research has begun to focus on restoring mitochondrial function and reducing oxidative stress. In IFNγ-driven myositis mice, excessive ROS production sustained muscle weakness and metabolic disruption. Treatment with NAC markedly improved muscle performance and restored mitochondrial structure and function (16). Consistent results were also observed in a passive transfer model of anti-SRP-induced necrotizing myopathy, in which NAC partially reversed oxidative stress and mitigated the impairment of mitochondrial OXPHOS (66). Clinically, NAC has been widely used as an antidote for acetaminophen overdose (95) and as a mucolytic/antioxidant agent in chronic airway diseases (96), raising expectations for its potential application in IIMs. Additionally, a series of mitochondria-targeting agents has been shown to have protective effects across patient-derived cells and animal models. Mitochonic acid-5 (MA-5) was shown to increase cellular ATP production and enhance the survival of fibroblasts derived from patients with mitochondrial diseases under stress-induced conditions (97). MA-5 also suppressed excessive ROS generation in a murine model of IBM (98). Moreover, the mitochondria-penetrating peptide Szeto–Schiller-31 (SS-31) was shown to restore mitochondrial ATP production and reduce oxidative stress in the muscles of aged and disuse-atrophy rodents (99). Similarly, the mitochondria-targeted ubiquinone derivative MitoQ was found to attenuate oxidative stress, thus improving mitochondrial respiratory function in mouse models of amyotrophic lateral sclerosis. A randomized controlled trial by Rossman et al. suggested that MitoQ might hold promise for treating age-related vascular dysfunction by reducing mitochondrial ROS levels (100). However, its therapeutic relevance to IIMs still needs further investigation (101, 102). Additionally, the natural antioxidant methyl 3,4-dihydroxybenzoate (MDHB) was shown to exert therapeutic effects in experimental autoimmune myositis mice. By reducing ROS and mitochondrial superoxide levels, it restored mitochondrial membrane potential and ATP levels and consequently alleviated muscle weakness (103). In a murine model of PM and an *in vitro* myotube system, the glucagon-like peptide-1 receptor (GLP-1R) agonist PF1801 downregulated the mitochondrial protein PGAM5 and suppressed FASLG-induced ROS in C2C12 myotubes, thus limiting necroptosis. *In vivo*, PF1801 improved muscle function and reduced inflammation in a mouse model (104). In addition, AMPK, which is one of the targets of metformin (105), was found to delay ageing through AMPK-mediated autophagic clearance and increased resistance to stress (106). The activation of AMPK was also shown to increase glucose uptake and promote mitochondrial biogenesis in cells and animal models (107, 108). This positions the AMPK pathway as a potential therapeutic target for restoring metabolic homeostasis in IIMs.

Mitochondrial transplantation has emerged as a promising regenerative approach (109). Kim et al. transplanted mitochondria isolated from human umbilical cord mesenchymal stem cells (PN-101) into patient-derived myoblasts and murine models of myositis. This intervention restored mitochondrial protein expression and ATP production while reducing inflammation in muscle tissues. (110). Encouragingly, a phase 1/2a trial (ClinicalTrials.gov identifier NCT04976140) in refractory PM and DM demonstrated preliminary safety and clinical improvement, with dose-limiting toxicity and 12-week International Myositis Assessment and Clinical Studies Group Total Improvement Score as the primary endpoint.

However, forced mitochondrial activation may be detrimental under inflammatory conditions. Basualto–Alarcón et al. showed that IIM muscle cells retained the capacity to increase oxidative metabolism, but forcing mitochondrial respiration led to increased oxidative stress and reduced viability. This raises the possibility that the downregulated mitochondrial activity observed in IIM may reflect an adaptive response to limit cellular damage (111). In this context, mitochondrial modulation requires fine-tuning rather than indiscriminate activation. Interventions that limit ROS, improve mitochondrial quality control, or support ATP production may be more suitable than simply activating mitochondrial. These considerations remain to be validated in disease-relevant models and clinical studies.

Moreover, sirolimus, an mTOR inhibitor with both immunomodulatory and metabolic effects, has been evaluated in a phase 2b randomized trial in IBM. Although no improvement was observed in muscle strength, several functional outcomes showed modest benefits, including 6-minute walking distance and respiratory capacity (112). Quantitative MRI analyses further suggested a slower progression of muscle fat replacement and atrophy under sirolimus treatment. However, phosphorus magnetic resonance spectroscopy demonstrated that abnormalities in high-energy phosphate metabolism remained largely unchanged (113). This indicates that sirolimus may not directly restore impaired muscle energy metabolism.

In addition to targeted molecular strategies, several metabolic supplementation trials have explored broader energy support approaches. A multicentre randomized controlled trial in PM/DM patients revealed that BCAA supplementation had no significant effect on muscle strength improvement, as evaluated by manual muscle testing (MMT), or on overall clinical response. However, it was partly effective at enhancing dynamic repetitive muscle functions (114). Additionally, Buzkova et al. performed omics in muscles from IBM patients and proposed Cr as a novel treatment target (115). However, another study by Solis et al. revealed that 12 weeks of Cr supplementation in JDM patients did not improve muscle function, as assessed by physical tests such as muscle strength measurements and timed functional tasks (116). This suggests that the therapeutic efficacy of Cr still needs further investigation.

Given their antioxidant properties, antioxidant strategies may be particularly beneficial in settings characterized by excessive ROS production and oxidative stress. Comparatively, SS-31 may be more relevant in conditions with impaired mitochondrial quality control, especially at the level of defective mitophagosome formation (117). In addition, MA-5 facilitates ATP synthase supercomplex formation and supports ATP generation even when electron transport chain is compromised (118). Therefore, it may be particularly beneficial in settings of impaired ATP production. However, current evidence does not yet support a state-specific therapeutic selection framework. Further studies are required to determine whether such context-dependent strategies can be translated into clinical practice.

Collectively, these studies—from cellular and animal models to early clinical trials—underscore growing interest in pharmacologic interventions that target energy metabolic abnormalities, especially mitochondrial injury and oxidative imbalance. Although the results remain preliminary, this expanding body of evidence highlights energy metabolism as a promising therapeutic axis in IIMs and warrants future mechanism-based clinical translation.

### 3.2 Nonpharmacological treatments

In addition to drug therapy, nonpharmacological approaches represent equally important components of comprehensive IIM management, which could benefit both metabolism and overall patient well-being. Among these, exercise therapy was one of the most effective interventions. Nemec et al. demonstrated that a six-month exercise regimen significantly ameliorated lipid metabolism dysregulation and improved energy utilization in muscle cells (22). Additionally, in patients with early-onset IIMs, high-intensity interval training (HIIT) has been shown to provide superior benefits over moderate-intensity exercise in increasing aerobic capacity and stimulating metabolic adaptations. This form of training enhances mitochondrial biogenesis and ATP production, which are fundamental processes for restoring energy balance in affected muscles (119). By proteomics analysis and immunohistochemical staining, Munters et al. reported that 12 weeks of endurance exercise in PM/DM patients might activate the phosphorylation metabolic pathway and promote muscle growth in diseased muscles (120). Boehler also demonstrated that endurance exercise improved mitochondrial function and slowed disease progression. This effect was associated with reduced expression of the harakiri (*HRK*) and Toll-like receptor 7 (*TLR7*) genes (34). In a chloroquine (CQ)-induced sIBM rat model, resistance exercise alleviated Aβ accumulation and enhanced mitochondrial biogenesis, dynamics, and autophagy. Collectively, these effects improved mitochondrial function and thus ameliorated CQ-induced muscle impairment (121). In addition to the real-time benefits, exercise was also demonstrated to have long-term effectiveness. Talotta et al. reported that a 12-week endurance training program significantly improved activities of daily living (ADL), maximal oxygen uptake (VO_2_max), and the physical function and vitality domains of the 36-item Short Form Health Survey (SF-36) in patients with IIMs compared with controls. These benefits in terms of muscle performance and general health were maintained at one year, which underscores the long-term effectiveness of exercise (122).

Nutritional interventions constitute another cornerstone of nonpharmacological management. Anti-inflammatory diets such as the Mediterranean diet, which is rich in omega-3 fatty acids, antioxidants and vitamin D, have been shown to reduce systemic inflammation and improve muscle function in immune-mediated diseases (123). Additionally, a ketogenic diet is theoretically capable of reducing inflammation, improving cellular bioenergetics, and ameliorating mitochondrial dysfunction. In a 52-year-old woman with progressive IBM, clinical improvement and attenuation of muscle wasting were observed following the adoption of a ketogenic diet (124).

**Figure 4:**
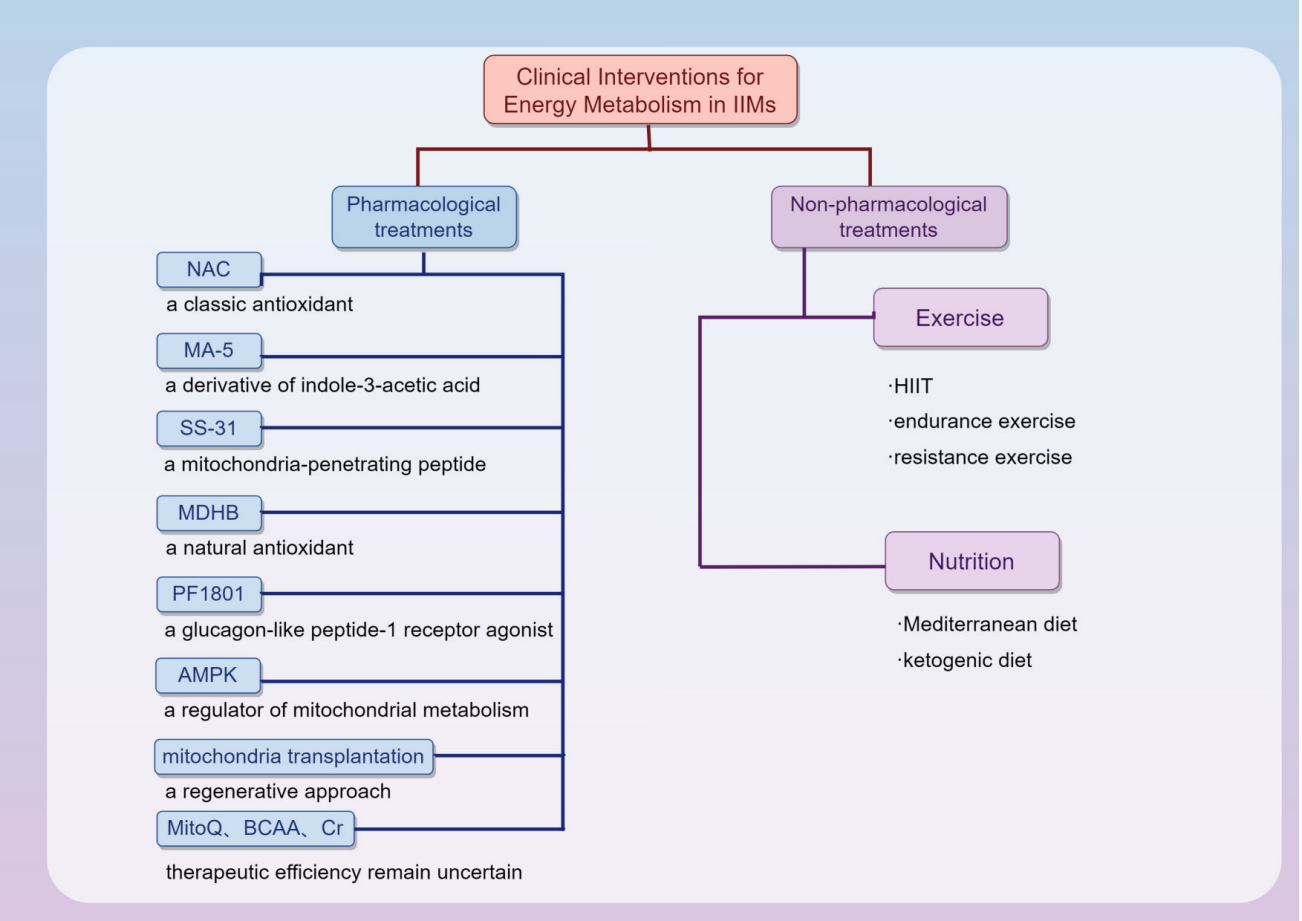
Potential therapeutic strategies targeting energy metabolism abnormalities in IIMs. Pharmacological approaches include N-acetylcysteine (NAC), Mitochonic acid-5 (MA-5), Szeto–Schiller-31 (SS-31), methyl 3,4-dihydroxybenzoate (MDHB), PF1801, AMP-activated protein kinase (AMPK) and mitochondria transplantation. Non-pharmacological interventions include exercise modalities, such as high-intensity interval training (HIIT), endurance, and resistance training, as well as dietary interventions, including Mediterranean and ketogenic diets.

Nonpharmacological treatments such as therapeutic exercise and dietary interventions play crucial roles in managing IIMs. When integrated with pharmacological therapies, these nonpharmacological strategies form a holistic approach to managing IIMs, which addresses both immunity and metabolism to improve patient outcomes. Personalized adaptation of these interventions, with physicians strengthening patient education and providing appropriate guidance, is essential to maximize therapeutic efficacy and patient benefit. A summary of pharmacological and non-pharmacological interventions is provided in **Figure 4**.

## 4 Discussion

In IIMs, accumulating evidence suggests that energy metabolism disturbances and immune inflammation interact to drive disease progression and muscle injury. Mechanistically, mitochondrial dysfunction and oxidative stress constitute the core of this process. Mitochondrial dysfunction is characterized by defective biogenesis, impaired mitophagy, and excessive ROS production. These alterations not only compromise the cellular energy supply but also activates inflammatory cascades through the cGAS–STING and type I interferon pathways. In parallel, metabolic reprogramming in glucose, lipid, and amino acid metabolism—particularly PKM2-driven glycolysis, mTOR/AMPK dysregulation, and tryptophan–kynurenine pathway activation—further amplifies immune signalling and tissue damage. Meanwhile, a growing number of biomarkers related to immunometabolic dysregulation in IIM have also been identified. These markers can be broadly categorized into exploratory biomarkers including omics-derived metabolic signatures and candidate pathway-related molecules, and clinically associated markers, such as urinary creatine-related indices and COX-deficient fibers, which may help for disease monitoring or prognostic assessment. Collectively, these findings highlight immunometabolic imbalance as an additional layer contributing to IIM pathogenesis. This extends beyond conventional inflammation-dominated mechanisms. However, the causal relationship between metabolic dysfunction and immune activation remains incompletely defined. Current evidence is largely derived from cross-sectional omics analyses or experimental models. This limits the ability to determine causality. It remains unclear whether metabolic alterations act as primary drivers, secondary adaptations, or context-dependent responses to inflammation.

Substantial heterogeneity exists in metabolic alterations across IIM subtypes (**Supplementary Table 1**). Current evidence does not support a uniform metabolic pattern. For example, enhanced glycolysis is observed in DM and PM, whereas sIBM shows downregulation of key glycolytic enzymes. TCA cycle activity exhibits inconsistent alterations across studies, even within the same subtype, likely reflecting differences in disease stage, tissue context, and experimental systems. Mitochondrial abnormalities further range from interferon-associated injury in DM to degenerative structural defects in IBM. Overall, these alterations exhibit clear subtype-specific features, which complicate direct comparisons and limit the establishment of a unified metabolic model.

Current therapeutic research targeting energy metabolism abnormalities in IIM primarily focuses on mitochondrial dysfunction and oxidative stress. Despite the growing number of mitochondria-directed strategies, a substantial translational gap persists. Most antioxidant and mitochondrial-modulating interventions have been validated mainly in murine models or patient-derived cell systems. However, these experimental systems may not fully recapitulate the sources of ROS, the inflammatory microenvironment, or the chronic immune activation observed in human IIM. In addition, currently available antioxidants lack large-scale, long-term phase III clinical trials. Key clinical parameters such as safety, optimal dosing, administration routes, and sustained efficacy across diverse IIM subsets remain inadequately defined. Mitochondrial transplantation using PN-101 represents a relatively advanced translational milestone, having progressed to early-phase clinical evaluation. Nevertheless, significant challenges remain, including standardization of mitochondrial isolation and delivery, durability of engraftment, potential immunogenicity, and cost-effectiveness in chronic autoimmune settings. Overall, although targeting mitochondrial dysfunction is a promising therapeutic direction, robust clinical validation and mechanistic refinement are still required before routine clinical integration.

Future research should aim to delineate how metabolic remodelling evolves across disease stages and cellular compartments and to identify reliable metabolic biomarkers for patient stratification and treatment response. Longitudinal and cell type-specific analyses are needed to clarify whether metabolic dysfunction precedes, parallels, or follows immune activation and how these interactions contribute to chronicity and treatment resistance. Integrative multiomics approaches—combining metabolomics, transcriptomics, proteomics, and spatial imaging—will be essential for constructing a comprehensive atlas of immunometabolic remodelling in IIMs. Importantly, such efforts should incorporate subtype-specific frameworks to account for the marked heterogeneity observed across IIM subgroups. The incorporation of metabolic profiling into clinical assessments could enable dynamic disease monitoring and facilitate precision, stratified treatment strategies. However, translating these approaches into clinical practice will require rigorous validation of candidate biomarkers and standardized assessment strategies. This may ultimately improve the prediction of therapeutic responsiveness to metabolic or immunomodulatory interventions. Finally, viewing IIMs through the lens of immune–metabolic miscommunication may provide additional therapeutic perspectives, complementing conventional immunosuppressive strategies and supporting efforts to restore cellular energy homeostasis and functional recovery.
