# Supplementary material for: Energy metabolism dysregulation in idiopathic inflammatory myopathies: mechanisms and therapeutic implications
Source: Front Immunol. 2026 Apr 17;17:1781434. doi: 10.3389/fimmu.2026.1781434 (PMC13132830; doi:10.3389/fimmu.2026.1781434)
Supplement: Supplementary file 1 [file Table1.docx]

**Table S1. Subtype-specific metabolic abnormalities across IIMs**

| **Metabolic category** | **DM/JDM** | **PM** | **IBM** | **IMNM** | **ASyS** |
| --- | --- | --- | --- | --- | --- |
| **Mitochondrial dysfunction & oxidative stress** | | | | | |
| **mtDNA** | mtDNA variants (D-loop) (27) | mtDNA variants (D-loop) (27) | mtDNA deletions/rearrangements (25,26) | ND | ND |
| **OXPHOS** | ↓genes of COX, NDUF and ATP synthase (28); ↑OXPHOS gene signatures (32) | ↓ (29) | ↓NADH dehydrogenase 2 (31); ↑OXPHOS pathways (33) | ND | ↑ (33) |
| **Mitochondrial structure** | ↓mitochondrial membrane potential (34); dysregulated mitochondria inner-membrane proteins (37, 43); | ↓mitochondrial membrane potential (34); dysregulated mitochondria inner-membrane proteins (37) | dysregulated mitochondria inner-membrane proteins (37, 43); disorganized cristae (43); ↓mitochondrial membrane potential, impaired mitochondrial elongation (44); | ND | ND |
| **Mitophagy** | ND | ND | ↓LC3B-II, p62 (deficient initiation) (44); ↑LC3, p62 (impaired degradation) (54), BNIP3↑ (55), ↑p-S65-Ub (56), ↓autophagosome, ↓autophagy-related genes (57) | ↑ degenerated mitochondria (58) | ND |
| **ROS / oxidative stress** | ↑ (16, 62, 63, 65) | ND | ND | ↑ (66) | ND |
| **Glucose metabolism** | | | | | |
| **Glycolysis** | ↑PKM2 (20) | ↑PKM2 (20) | ↓GAPDH, LDHA, GPD1, PYGM (70) | ND | ND |
| **TCA cycle** | ND | ↓CS, MDH (21) | ↑TCA cycle intermediates (e.g., succinate, fumarate, citrate) and anaplerotic amino acids (e.g., glutamate, aspartate, asparagine) (56); ↓TCA cycle byproducts (e.g., succinate, glutamate) (71) | ND | ND |
| **Lipid metabolism** | | | | | |
| **Serum level** | ↑TG, ↓HDL-C (78); ↑palmitic acid (76); sphingolipids↑, ↑lysophospholipids (79); ↑ACs and ceramides (80) | ↑TG, ↓HDL-C (77); ↑palmitic acid (76) | ND | ND | ND |
| **Muscle level** | ↓oxidative and non-oxidative lipid metabolism, ↑ATGL (22) | ↓oxidative and non-oxidative lipid metabolism, ↑ATGL (22) | ↑free cholesterol, lipoprotein receptors (81) | ↓oxidative and non-oxidative lipid metabolism, ↑ATGL (22) | ND |
| **Amino acid metabolism** | | | | | |
| **Serum and muscle level** | ↓BCAAs (86); ↓phenylalanine and tryptophan (87) | ↓BCAAs (86) | ↑anaplerotic amino acids (56) | ND | ↓BCAAs (86) |
| **Urinary level** | ↑Cr, ↑taurine, ↑glycine (88); Cr/creatinine ratio↑ (89) | ↑Cr, taurine, glycine (88) | ND | ND | ND |
| **Trp–Kyn pathway** | ↑ (23) | ND | ND | ND | ND |

Abbreviations: IIMs, idiopathic inflammatory myopathies; DM, dermatomyositis; JDM, juvenile dermatomyositis; PM, polymyositis; IBM, inclusion body myositis; IMNM, immune-mediated necrotizing myositis; ASyS, antisynthetase syndrome; mtDNA, mitochondrial DNA; ND, not defined; OXPHOS, oxidative phosphorylation; COX, cytochrome c oxidase; ROS, reactive oxygen species; PKM2, pyruvate kinase M2; GAPDH, glyceraldehyde-3-phosphate dehydrogenase; LDHA, lactate dehydrogenase A; GPD1, glycerol-3-phosphate dehydrogenase 1; PYGM, glycogen phosphorylase; TCA, tricarboxylic acid; CS, citrate synthase; MDH, malate dehydrogenase; TG, triacylglycerol; HDL-C, high density lipoprotein cholesterol; ACs, acylcarnitines; ATGL, adipose triglyceride lipase; BCAAs, branched-chain amino acids; Cr, creatine; Trp–Kyn, tryptophan-kynurenine.
